# Melatonin Alleviates Chromium Toxicity in Maize by Regulating Polyamine Metabolism and Enhancing Antioxidant Activity

**DOI:** 10.3390/plants15101434

**Published:** 2026-05-08

**Authors:** Juanjuan Ma, Ke Feng, Guo Wang, Xinru Wang, Leyong Feng, Jianhong Ren

**Affiliations:** 1 Maize Research Institute, Shanxi Agricultural University, Xinzhou 034000, China; sxaumjj@163.com; 2 College of Agriculture, Shanxi Agricultural University, Taigu 030800, China; sxau_fk@126.com (K.F.); sxau_wg@126.com (G.W.); sxau_wxr@126.com (X.W.); 3 College of Life Sciences, Shanxi Agricultural University, Taigu 030800, China

**Keywords:** maize, chromium, melatonin, polyamine metabolism, redox homeostasis

## Abstract

Chromium (Cr) contamination leads to the accumulation of Cr in crops, thereby posing a significant threat to food security and human health. It is essential to comprehend the mechanisms underlying Cr toxicity and to develop effective mitigation strategies to ensure healthy crop growth. Melatonin (MT), a multifunctional regulatory molecule, plays a pivotal role in the response of plants to heavy metal stress. This study is designed to investigate the underlying mechanisms through which exogenous application of MT mitigates the toxicity of Cr stress in maize seedlings. The findings of the study indicate that under Cr stress conditions, treatment with MT significantly decreased the Cr concentrations in the roots and leaves of maize, with reductions of 22% and 28.5%, respectively. Concurrently, MT demonstrated effectiveness in alleviating the toxic effects induced by Cr exposure, as evidenced by substantial improvements in the leaf area, chlorophyll content, and photosynthetic rate, which increased by 40.3%, 47.7%, and 64.8%, respectively. This led to a 42.2% increase in the total dry weight of maize. Further analysis indicates that MT modulates the antioxidant system, thereby reducing the production of reactive oxygen species and reducing membrane lipid damage associated with Cr toxicity. Moreover, MT upregulates the expression and activity of enzymes involved in polyamine synthesis while simultaneously inhibiting the activity of polyamine-degrading enzymes, leading to a 38% increase in total polyamine content. This study has enhanced our understanding of the mechanisms through which melatonin alleviates chromium toxicity in crops and has provided a theoretical foundation for its sustainable application in agricultural production.

## 1. Introduction

Heavy metal pollution constitutes a significant environmental challenge of global concern. The ongoing processes of industrialization and urbanization have contributed to a continuous rise in the levels of heavy metals in the environment [1]. Chromium (Cr) is recognized as one of the most detrimental heavy metals. Over the past five decades, emissions of Cr have surpassed 30,000 tons globally, resulting from both natural sources and anthropogenic activities [2]. Substantial amounts of Cr are introduced into agricultural land through natural phenomena such as volcanic eruptions, as well as through human activities including the use of paint, operations in the leather industry, and emissions from industrial smoke and dust, leading to significant soil contamination [3,4]. Research has demonstrated that the accumulation of Cr in the organs of plants can impede normal growth by disrupting water and mineral nutrient uptake, diminishing photosynthetic efficiency, and disturbing metabolic equilibrium [5]. Furthermore, the Cr accumulated in crops has the potential to enter the human body via the food chain, leading to various diseases, including lung and gastric cancers, thereby posing a significant threat to human health [6]. Considering the detrimental impact of Cr pollution on both crop development and human health, it is urgent to formulate effective strategies to reduce the accumulation of Cr in crops and mitigate its toxic effects.

In recent years, a variety of remediation technologies have been developed to mitigate heavy metal contamination in agroecosystems. These include physicochemical amendments, phytoremediation, and microbe-assisted approaches, as well as emerging strategies such as genetic engineering and synthetic biology [7]. Increasing attention has been directed toward enhancing the intrinsic tolerance of plants to heavy metals. Among these strategies, exogenous regulatory molecules represent a promising and practical approach, as they can directly modulate plant physiological responses, thereby enabling plants to more effectively cope with heavy metal stress [8].

Melatonin (MT) is a compound extensively found in both plants and animals, first identified in plants in 1995 [9,10]. As a multifunctional regulatory molecule, MT is integral to numerous physiological processes, including seed germination, root development, photosynthesis, flowering, and leaf senescence [11]. Notably, MT is also pivotal in enhancing plant tolerance to a variety of environmental stresses, including salinity, drought, and heavy metal exposure [12]. Recent research has examined the potential of exogenous MT to mitigate the detrimental impacts of chromium toxicity in plants. For instance, exogenous MT has been shown to significantly enhance the growth and yield of wheat in Cr-contaminated soil by reducing Cr uptake and toxicity [13]. The study conducted by Farouk and Al-Amri [14] demonstrated that foliar application of MT can alleviate Cr-induced senescence in marjoram by maintaining redox equilibrium, promoting osmotic regulation, and preserving cellular ultrastructure stability. Additionally, MT has been shown to enhance photosynthesis through the regulation of carotenoid synthesis, thereby diminishing the detrimental effects of Cr on plants [15]. However, the underlying mechanisms by which MT alleviates Cr stress require further elucidation.

Polyamines are a class of aliphatic compounds characterized by the presence of two or more amine groups, primarily encompassing putrescine (Put), spermidine (Spd), and spermine (Spm) [16]. Research indicates that polyamines are involved in a range of physiological processes, including cell division and differentiation, embryonic development, flower bud differentiation, leaf growth, fruit ripening, and senescence [17]. Furthermore, elevated polyamine accumulation in plants has been shown to improve their resilience against various abiotic stresses, including drought, salinity, temperature extremes, and heavy metal toxicity [18]. For example, exogenous application of polyamines can reduce Cr accumulation, enhance antioxidant enzyme activity, improve photosynthetic efficiency, and maintain hormonal balance, thereby increasing rice’s tolerance to Cr toxicity [19]. Additionally, polyamines can mitigate the growth inhibition caused by Cr toxicity in maize by activating antioxidant defense mechanisms, reducing cellular oxidative damage, and enhancing membrane structural stability [20]. Under conditions of mild drought, polyamines have also been observed to inhibit stomatal closure induced by abscisic acid, thereby preserving the photosynthetic capacity of plants [21].

Upon the absorption of Cr by plants, there is a significant induction of reactive oxygen species (ROS). These ROS cause oxidative damage to proteins, nucleic acids, and lipids in plants. Furthermore, they disrupt the cell membrane, compromising its structural integrity and resulting in an imbalance in the redox state, as well as the leakage of intracellular substances [22,23]. When exposed to oxidative stress, plants initiate antioxidant defense systems that include enzymatic antioxidants—such as superoxide dismutase (SOD), catalase (CAT), and peroxidase (POD)—alongside non-enzymatic compounds, including glutathione (GSH) and ascorbic acid (AsA) [24]. Nevertheless, under heavy metal stress conditions, while plants may enhance the activity of certain antioxidant enzymes and increase the levels of non-enzymatic antioxidants, the complete elimination of excessive ROS and the mitigation of oxidative damage remain challenging. The application of exogenous regulatory substances can further stimulate the antioxidant defense system, thereby facilitating a more effective reduction in damage caused by ROS [23,25].

Maize (*Zea mays* L.) is an essential global food crop, serving as a source of biomass energy and feed, and is integral to sustaining population growth and industrial development [26]. However, the presence of heavy metal pollutants in soil poses a significant threat to maize production [27]. Cr in environmental systems predominantly occurs in two valence forms, namely tri-valent [Cr(III)] and hexavalent [Cr(VI)]. Among these, Cr(VI) exhibits significantly higher toxicity and is categorized as a Group 1 human carcinogen by the International Agency for Research on Cancer [28]. Consequently, this study focuses on hexavalent Cr as the subject of investigation. Previous research has demonstrated that exogenous MT enhances plant tolerance to Cr by activating antioxidant defense mechanisms and promoting osmotic regulation [14]. Furthermore, polyamines are crucial in maintaining redox homeostasis and supporting plant growth and development under Cr stress [20]. Growing evidence indicates that under abiotic stress conditions, including salinity and low temperatures, exogenous MT facilitates polyamine accumulation in plants by upregulating transcription factors such as ZAT2/6/12 and CsMYB44. This process is intricately linked to improved stress tolerance in plants [29,30,31]. Building upon these findings, we propose the hypothesis that MT may mitigate the toxicity associated with Cr stress in maize by modulating polyamine metabolism. Consequently, this study utilized maize as the experimental material to investigate the accumulation of Cr and MT within the plant, alongside examining alterations in polyamine metabolism, redox homeostasis, and plant biomass. This study investigates how MT reduces Cr stress, offering a basis for using external MT to lessen Cr’s harmful impact on corn growth.

## 2. Results

### 2.1. Effects of MT and Cr on Growth Parameters of Maize

**Figure 1:**
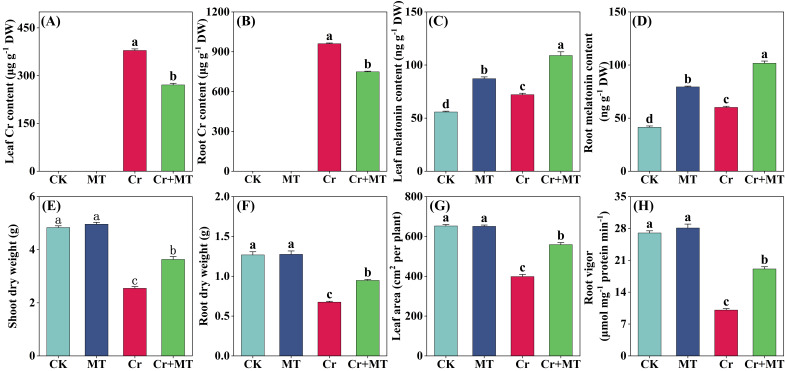
Effects of melatonin and chromium treatment on leaf/root Cr content (**A**,**B**); leaf/root MT content (**C**,**D**); shoot dry weight (**E**); root dry weight (**F**); leaf area (**G**); root vigor (**H**) of maize. CK: Control; MT: Melatonin; Cr: Chromium; Cr + MT: Melatonin + Chromium. Values are means ± SE (*n* = 4). The distinct lowercase letters along the horizontal axis denote statistically significant differences among the various treatment groups (*p* ˂ 0.05).

After a 7-day exposure to Cr stress, a significant accumulation of Cr was observed in maize plants. Exogenous MT markedly reduced this accumulation. Specifically, in comparison to the treatment with Cr alone, MT application resulted in a reduction in Cr content by 28.5% in the leaves and 22% in the roots. Furthermore, under both normal and Cr stress conditions, exogenous MT enhanced the endogenous MT levels in the roots and leaves, with a more pronounced increase observed under Cr stress conditions (Figure 1A–D). These findings suggest that exogenous MT effectively mitigates Cr uptake in maize plants and its translocation to shoots parts. Under standard growth conditions, MT supply did not significantly influence the dry biomass of the maize plants (Figure 1E,F). However, when exposed to Cr stress, the dry biomass of maize shoots and roots significantly decreased by 40.4% and 42.7%, respectively. Notably, MT supply effectively alleviated the inhibitory impact of Cr stress on dry biomass accumulation in maize. Concurrently, MT treatment markedly enhanced the leaf area and root vitality of maize seedlings under Cr stress (Figure 1G,H). Overall, the exogenous application of MT effectively reduces Cr accumulation in maize plants and alleviates the adverse effects of Cr stress on plant growth.

### 2.2. Effects of MT and Cr on Photosynthetic of Maize

**Figure 2:**
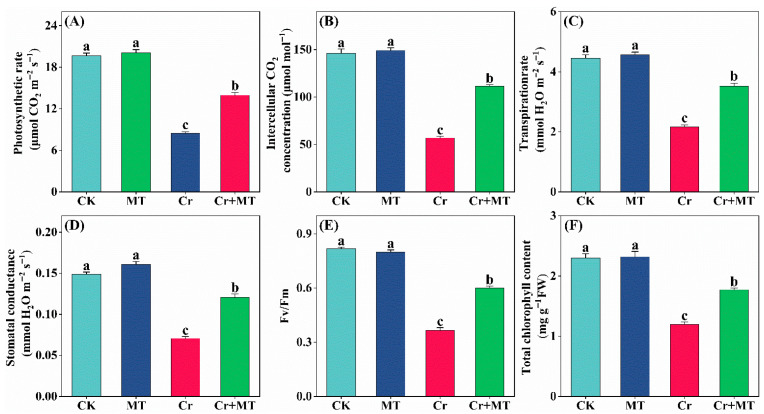
Effects of melatonin and chromium treatment on photosynthetic rate (Pn, (**A**)); intercellular CO_2_ concentration (Ci, (**B**)); transpiration rate (Tr, (**C**)); stomatal conductance (Gs, (**D**)); Fv/Fm (**E**); total chlorophyll content (**F**) of maize. CK: Control; MT: Melatonin; Cr: Chromium; Cr + MT: Melatonin + Chromium. Values are means ± SE (*n* = 4). The distinct lowercase letters along the horizontal axis denote statistically significant differences among the various treatment groups (*p* ˂ 0.05).

To investigate the potential mechanisms through which MT mitigates the inhibitory effects of Cr stress on maize growth, this study examined variations in chlorophyll content, photosynthetic parameters (photosynthetic rate (Pn), stomatal conductance (Gs), intercellular CO_2_ concentration (Ci), and transpiration rate (Tr), and the maximum quantum efficiency of photosystem II (Fv/Fm)) in maize leaves subjected to different treatments (Figure 2). Under optimal conditions, applying MT externally did not significantly affect chlorophyll content, photosynthetic parameters, or Fv/Fm. In contrast, under Cr stress, there was a significant reduction in total chlorophyll content, photosynthetic parameters, and Fv/Fm, indicating substantial impairment of photosynthetic function. Notably, the application of exogenous MT significantly mitigated the decline in photosynthetic-related parameters. In comparison to the treatment with Cr stress alone, the MT + Cr treatment led to significant increases in total chlorophyll content, Fv/Fm, Tr, Gs, Ci, and Pn by 47.7%, 64.4%, 62.9%, 70.3%, 95.8%, and 64.8%, respectively. These findings suggest that MT effectively protects the photosynthetic machinery of maize leaves from Cr-induced damage, thereby sustaining photosynthetic productivity under Cr stress.

### 2.3. Effects of MT and Cr on the Metabolism of Endogenous Polyamines of Maize

**Figure 3:**
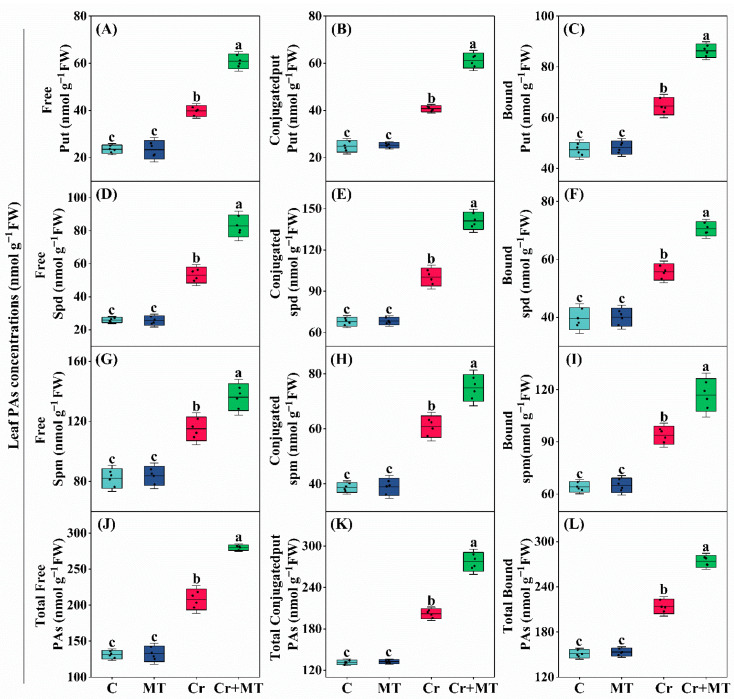
Effects of melatonin and chromium treatment on leaf polyamine (PAs) fractions in maize, including free, conjugated, and bound putrescine (Put, (**A**–**C**)); free, conjugated, and bound spermidine (Spd, (**D**–**F**)); free, conjugated, and bound spermine (Spm, (**G**–**I**)); free, conjugated, and bound PAs (**J**–**L**). CK: Control; MT: Melatonin; Cr: Chromium; Cr + MT: Melatonin + Chromium. Values are means ± SE (*n* = 4). The distinct lowercase letters along the horizontal axis denote statistically significant differences among the various treatment groups (*p* ˂ 0.05).

**Figure 4:**
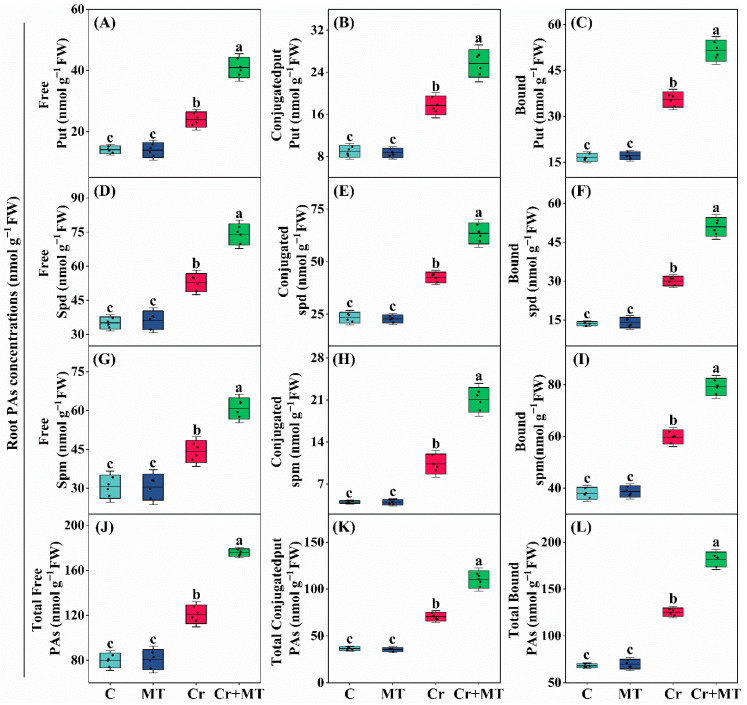
Effects of melatonin and chromium treatment on root polyamine (PA) fractions in maize, including free, conjugated, and bound putrescine (Put, (**A**–**C**)); free, conjugated, and bound spermidine (Spd, (**D**–**F**)); free, conjugated, and bound spermine (Spm, (**G**–**I**)); free, conjugated, and bound PAs (**J**–**L**). CK: Control; MT: Melatonin; Cr: Chromium; Cr + MT: Melatonin + Chromium. Values are means ± SE (*n* = 4). The distinct lowercase letters along the horizontal axis denote statistically significant differences among the various treatment groups (*p* ˂ 0.05).

**Figure 5:**
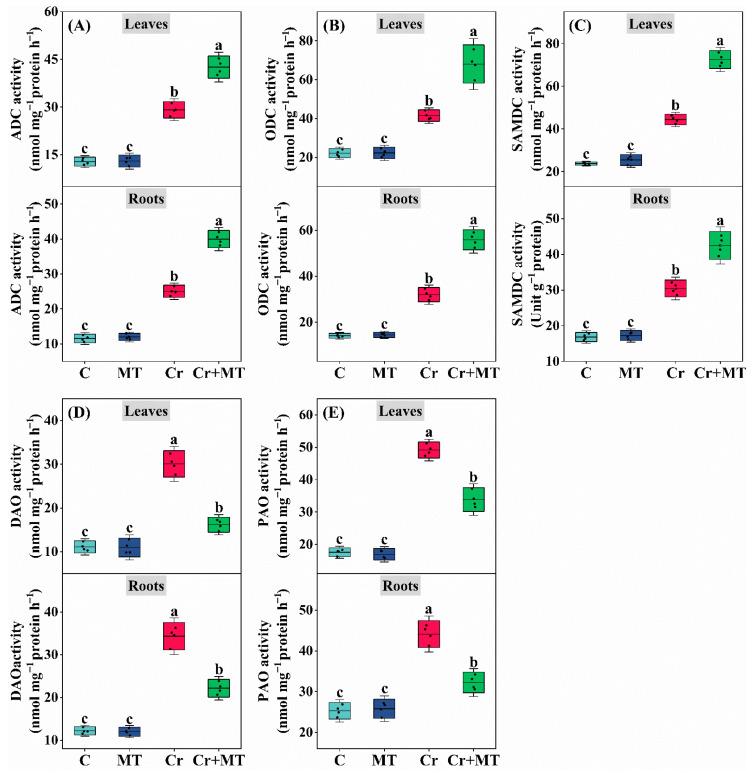
Effects of melatonin and chromium treatment on arginine decarboxylase (ADC, (**A**)), ornithine decarboxylase (ODC, (**B**)), S-adenosyl methionine decarboxylase (SAMDC, (**C**)), diamine oxidase (DAO, (**D**)), polyamine oxidase (PAO, (**E**)) activity in maize leaves and roots. CK: Control; MT: Melatonin; Cr: Chromium; Cr + MT: Melatonin + Chromium. Values are means ± SE (*n* = 4). The distinct lowercase letters along the horizontal axis denote statistically significant differences among the various treatment groups (*p* ˂ 0.05).

**Figure 6:**
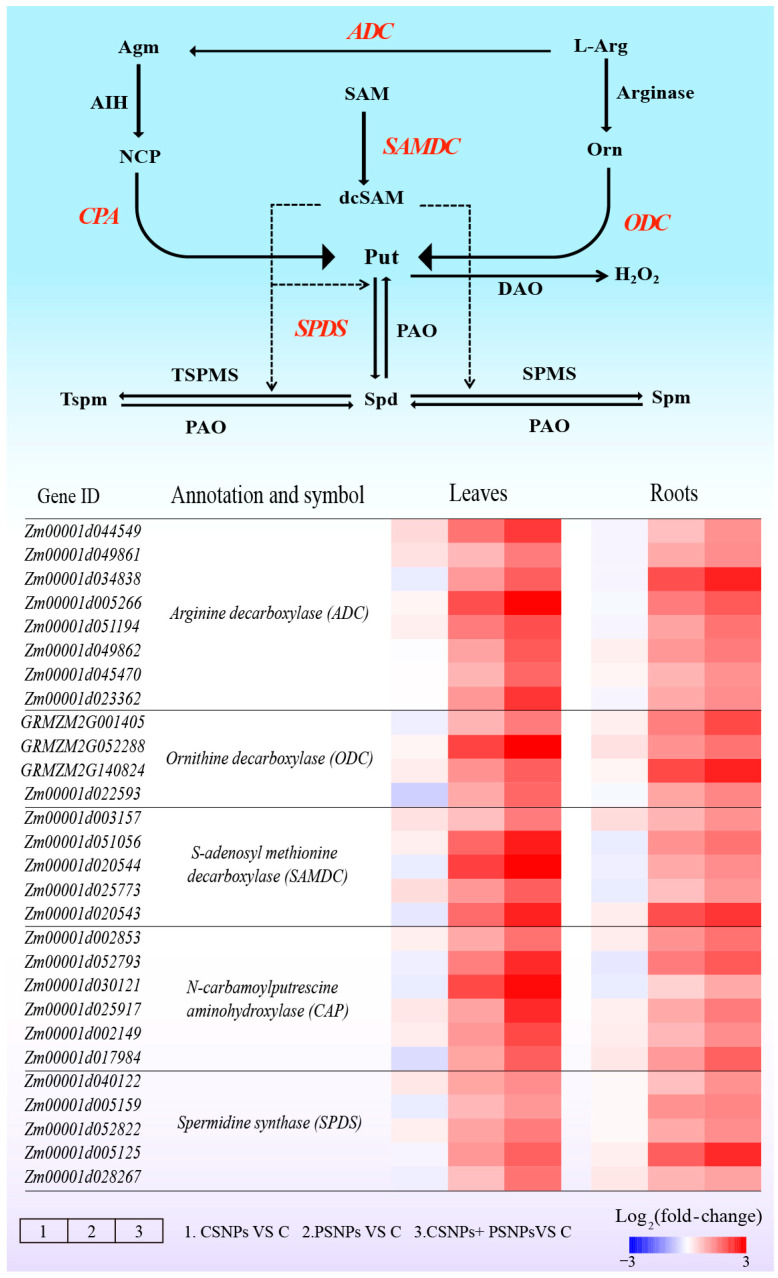
Effects of melatonin and chromium treatments on the expression of genes associated with polyamine biosynthesis in maize leaves and roots. CK: Control; MT: Melatonin; Cr: Chromium; Cr + MT: Melatonin + Chromium. The scale bar represents the log_2_ (fold-change) value.

To investigate whether the mechanism by which MT mitigates Cr stress in maize is linked to polyamine metabolism, changes in polyamine profiles were analyzed in seedlings exposed to different treatments. Under standard conditions, applying MT externally did not significantly influence the concentrations of different forms (including free, soluble-conjugated, and insoluble-bound) of Spd, Put, and Spm, in maize seedlings. However, under Cr stress, the levels of Spd, Put, and Spm in maize exhibited a significant increase, with further elevation observed following exogenous MT treatment (Figure 3 and Figure 4). Additionally, the analysis of enzyme activities related to polyamine synthesis revealed a similar trend; specifically, under Cr stress, the activities of arginine decarboxylase (ADC), ornithine decarboxylase (ODC), and S-adenosylmethionine decarboxylase (SAMDC) were significantly enhanced, with exogenous MT further augmenting the activities of these enzymes. For instance, when compared to the treatment with Cr alone, the application of exogenous MT resulted led to an enhancement in the activities of the enzymes ADC, SAMDC, and ODC in maize leaves by 46.2%, 63.3%, and 63.9%, respectively, and in roots by 59.7%, 39.6%, and 74.4% (Figure 5A–C). Conversely, under Cr stress conditions, there was a significant elevation in the activities of the polyamine-degrading enzymes diamine oxidase (DAO) and polyamine oxidase (PAO) in maize plants. However, the introduction of exogenous MT markedly suppressed this increase. Specifically, when compared to the Cr treatment alone, the MT + Cr treatment led to significant reduction in PAO and DAO activities in leaves by 46.2% and 31.1%, respectively, and in roots by 35.5% and 27.0%, respectively (Figure 5D,E). Furthermore, the study examined the expression levels of genes related to polyamine biosynthesis in maize roots and leaves, revealing that their trends were consistent with the activities of polyamine synthase and the polyamine content (Figure 6). In summary, polyamines potentially play a pivotal role in mitigating Cr toxicity in maize through the action of MT.

### 2.4. Effects of MT and Cr on Oxidative Stress

**Figure 7:**
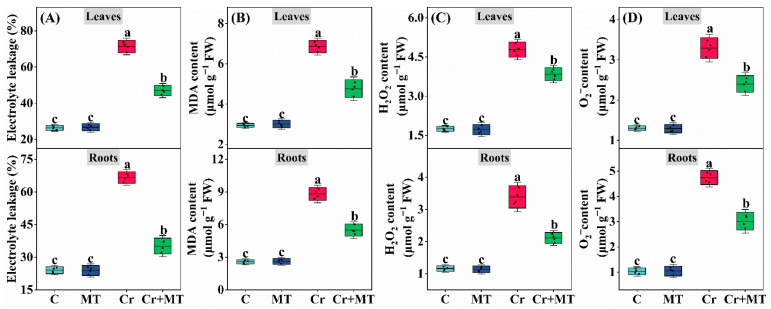
Effects of melatonin and chromium treatment on electrolyte leakage (**A**), malondialdehyde content (MDA, (**B**)), hydrogen peroxide content (H_2_O_2_, (**C**)), superoxide anion radical content (O_2_^−^, (**D**)) in maize leaves and roots. CK: Control; MT: Melatonin; Cr: Chromium; Cr + MT: Melatonin + Chromium. Values are means ± SE (*n* = 4). The distinct lowercase letters along the horizontal axis denote statistically significant differences among the various treatment groups (*p* ˂ 0.05).

Upon exposure of maize seedlings to Cr, there was a notable increase in the levels of hydrogen peroxide (H_2_O_2_) and superoxide anion (O_2_^−^) within the roots and leaves. These ROS are known for their redox toxicity, posing a threat to plant cellular integrity. Subsequent analyses demonstrated that Cr treatment markedly elevated malondialdehyde (MDA) content and relative conductivity, signifying substantial oxidative damage to the cells. Conversely, exogenous MT significantly alleviated oxidative damages. Compared to the Cr treatment alone, MT application resulted in a reduction in O_2_^−^ and H_2_O_2_ levels in the leaves by 27.1% and 19.6%, respectively, and in the roots by 36.4 and 37.7% (Figure 7C,D). Furthermore, MT significantly lowered the MDA content and Electrolyte leakage (Figure 7A,B). These findings suggest that MT plays a crucial role in ameliorating the Cr-induced phytotoxicity.

### 2.5. Effects of MT and Cr on the Antioxidant Defense System

**Figure 8:**
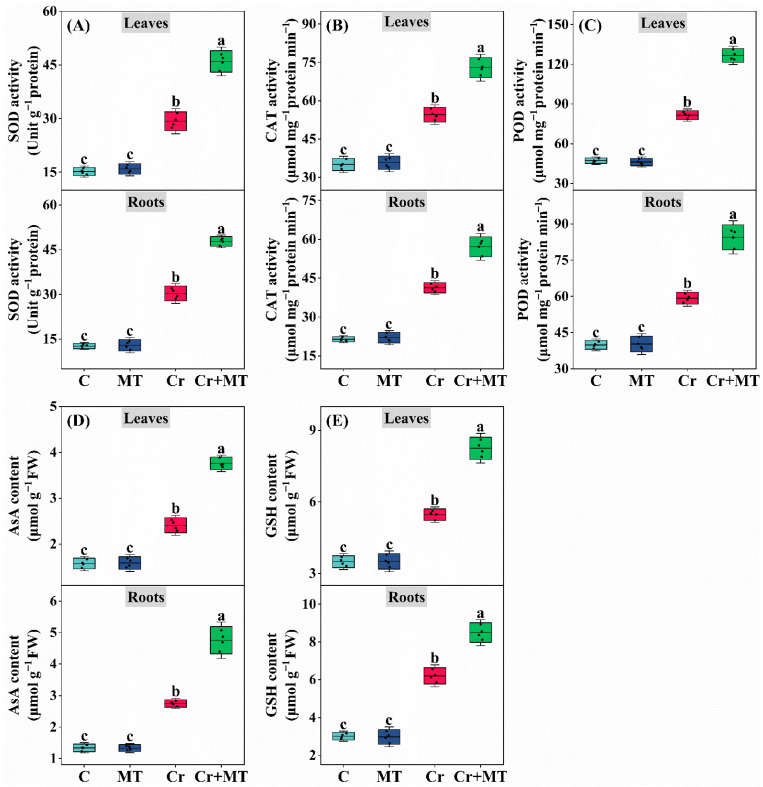
Effects of melatonin and chromium treatment on superoxide dismutase activity (SOD, (**A**)), catalase activity (CAT, (**B**)), peroxidase activity (POD, (**C**)), ascorbate content (AsA, (**D**)), reduced glutathione content (GSH, (**E**)) in maize leaves and roots. CK: Control; MT: Melatonin; Cr: Chromium; Cr + MT: Melatonin + Chromium. Values are means ± SE (*n* = 4). The distinct lowercase letters along the horizontal axis denote statistically significant differences among the various treatment groups (*p* ˂ 0.05).

**Figure 9:**
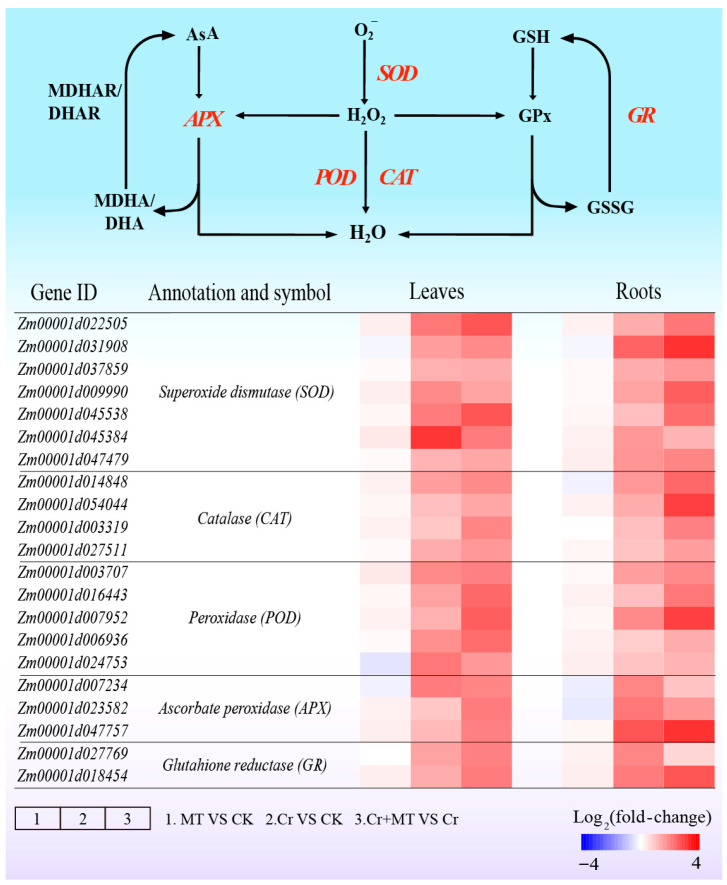
Effects of melatonin and chromium treatments on the expression of genes associated with biosynthesis of antioxidant enzymes in maize leaves and roots. CK: Control; MT: Melatonin; Cr: Chromium; Cr + MT: Melatonin + Chromium. The scale bar represents the log_2_ (fold-change) value.

To elucidate the critical function of MT in alleviating oxidative-reductive damage caused by Cr stress, we evaluated the activities of antioxidant enzymes in maize seedlings. The results indicated that exogenous MT significantly enhanced the activities of SOD, POD, and CAT under Cr stress conditions (Figure 8A–C). Specifically, compared to Cr treatment alone, MT application increased the activities of SOD, POD, and CAT by 57.1%, 55.3%, and 33.9%, respectively, in the leaves, and by 57.7%, 42.5%, and 38.2%, respectively, in the roots. In alignment with these changes in antioxidant enzyme activities, MT treatment also significantly elevated the levels of non-enzymatic antioxidants, namely AsA and GSH, in maize seedlings subjected to Cr stress (Figure 8D,E). Moreover, an analysis of the expression levels of genes associated with antioxidant enzymes revealed that exogenous MT significantly upregulated their expression under Cr stress conditions (Figure 9). These results suggest that MT activates the antioxidant system, thereby enhancing the clearance of ROS induced by Cr stress and alleviating oxidative damage.

## 3. Discussion

Cr contamination in agricultural soils presents significant risks to food security, ecosystems, and human health. Research indicates that once Cr is absorbed by plants, its accumulation within plant tissues can disrupt the antioxidant defense system, impair water and nutrient uptake, diminish photosynthetic activity, and disturb metabolic equilibrium, ultimately inhibiting plant growth [5,23]. This study found that under Cr stress conditions, the Cr concentration in maize increased markedly, resulting in significant growth inhibition (Figure 1). Previous research has indicated that the application of exogenous MT can alleviate the growth inhibitory effects of heavy metals on plants such as watermelon, wheat, and tomato [32,33,34]. In the current study, exogenous MT markedly increased dry matter accumulation in maize plants subjected to Cr stress and effectively alleviated the Cr-induced growth inhibition (Figure 1E,F). Notably, MT also substantially reduced the Cr accumulation in the leaves and roots of maize seedlings (Figure 1A,B). Previous studies have shown that MT can reduce heavy metal uptake and translocation in plants by regulating the expression of metal transport–related genes (such as *HMA2*, *IRT1*, and *Nramp5*) and by promoting the accumulation of cell wall components (including cellulose, hemicellulose, and pectin) [35,36,37]. The substantial decrease in Cr accumulation in the leaves and root observed in this study may be attributed to the modulation of these mechanisms.

Cr stress typically impairs photosynthesis, which is fundamental to the formation of plant biomass [38]. The present study demonstrates that MT can enhance photosynthetic activity, which is essential for maize’s resilience to chromium toxicity. Specifically, the application of MT resulted in significant increases in the Pn, Ci, Gs, and Tr under Cr stress (Figure 2). In alignment with prior research, MT has been shown to mitigate the photosynthetic inhibition induced by Cr stress through the regulation of stomatal conductance and intercellular carbon dioxide concentration [39]. Furthermore, MT promotes CO_2_ absorption by enhancing the activity of Rubisco, thereby sustaining photosynthesis in tomato leaves subjected to heat stress [40]. Chlorophyll content and maximum quantum efficiency of photosystem II serve as essential indicators of a plant’s capacity to absorb and convert light energy. Recent research has demonstrated that MT can enhance the concentration of photosynthetic pigments under salt stress conditions, thereby improving the efficiency of light energy absorption and transmission in rice [41]. Furthermore, exogenous MT has been demonstrated to restore the photosynthetic capacity of Chlamydomonas reinhardtii under Cr stress [15] and tobacco under nitrate stress [42] by preserving the integrity of thylakoid membranes, stabilizing the ultrastructure of chloroplasts, and inhibiting enzymes responsible for chlorophyll degradation. In the present study, exogenous MT markedly elevated the total chlorophyll content and the Fv/Fm in maize subjected to Cr stress, thereby improving their capacity for light energy absorption and conversion (Figure 2E,F). Collectively, these findings provide valuable insights into the mechanisms through which MT mitigates Cr-induced photosynthetic inhibition.

Polyamines play essential roles in regulating plant growth, developmental processes, and adaptation to abiotic stress conditions. Research has demonstrated that the synthesis of polyamines can be upregulated in citrus plants subjected to Cr stress [43]. Furthermore, the exogenous application of polyamines has been shown to mitigate Cr toxicity in maize by enhancing antioxidant enzyme activity, improving the photosynthetic apparatus, and maintaining hormonal equilibrium [19]. In the present study, the application of exogenous MT significantly elevated the levels of Put, Spd, and Spm in the roots and leaves of maize under Cr stress (Figure 3 and Figure 4). DAO and PAO are enzymes that decompose polyamines, resulting in the production of H_2_O_2_ [44]. This investigation revealed that exogenous MT significantly decreased the activities of these two enzymes, thereby promoting the accumulation of polyamines (Figure 5D,E). In terms of the mechanisms by which polyamines alleviate heavy metal stress, studies indicate that polyamines can inhibit the self-oxidation of metal ions, thereby preventing the generation of ROS. Additionally, they regulate the expression of antioxidant-related genes through transcriptional mechanisms or directly activate antioxidant enzymes, thereby enhancing the plant’s capacity to eliminate ROS [17]. In conclusion, MT markedly enhanced the accumulation of polyamines while concurrently diminishing the production of ROS, thereby improving the tolerance of maize seedlings to Cr stress.

The equilibrium between reactive ROS production and clearance is essential for plant growth, development, and stress resistance [45]. Under Cr stress, plants experience an overproduction of ROS, resulting in membrane lipid peroxidation and cellular damage [23]. Recent research indicates that MT can effectively mitigate oxidative damage induced by Cr, significantly decreasing the accumulation of ROS and malondialdehyde levels [46]. In this study, exogenous application of MT notably reduced the levels of O_2_^−^ and H_2_O_2_ under Cr stress, and decreased the content of the membrane damage marker MDA and electrolyte leakage (Figure 7). Comparable outcomes have been reported in Cr-stressed rapeseed [47], Chlamydomonas reinhardtii [15], and wheat [46]. To counteract oxidative damage caused by ROS, plants have developed intricate clearance systems, wherein both enzymatic and non-enzymatic antioxidants are pivotal. In marjoram plants, foliar application of MT activated antioxidant enzyme activity, reduced ROS accumulation, and effectively alleviated leaf damage induced by Cr [14]. Awan et al. [48] demonstrated that the application of MT enhances the expression of genes associated with antioxidant enzymes, while simultaneously reducing the accumulation and toxicity of ROS induced by cadmium in pearl millet. The findings of this study corroborate these results. Under Cr stress, the application of MT significantly upregulated the expression levels of SOD, CAT, and POD (Figure 9), which was consistent with the observed increase in enzyme activity (Figure 8A–C). Furthermore, GSH and AsA are critical compounds for the detoxification of ROS and may also chelate heavy metals within cells [49]. This study revealed that the exogenous application of MT elevated the concentrations of GSH and AsA in the leaves and roots of maize, thereby enhancing the plant’s capacity to eliminate ROS (Figure 8D,E). These findings align with previous research indicating that exogenous MT can increase the levels of AsA and GSH under Pb stress, subsequently reducing excessive ROS accumulation and mitigating oxidative damage [50]. Similar outcomes were reported in studies examining the role of MT in alleviating cadmium toxicity in juncea plants [51]. Collectively, these results suggest that the exogenous application of MT activates the antioxidant defense system and facilitates the clearance of excessive ROS, thereby restoring the redox balance in maize subjected to Cr stress. Recently, Ali et al. [52] elucidated that excessive levels of ROS can result in irreversible cellular damage, whereas regulated concentrations of ROS function as signaling molecules that facilitate physiological adjustments and adaptive responses in plants, thereby enhancing their resilience to environmental stressors. For example, reactive oxygen species are known to activate the mitogen-activated protein kinase (MAPK) signaling pathway, modulate downstream transcription factors (including bZIP, WRKY, and MYB), promote the expression of genes encoding detoxification enzymes, and increase the plant’s tolerance to heavy metal exposure [53]. This study demonstrates that MT alleviates Cr stress by activating the antioxidant defense system and promoting polyamine accumulation, thereby effectively reducing ROS accumulation and maintaining cellular redox homeostasis, which is crucial for the normal functioning of ROS as signaling molecules.

In conclusion, this study elucidates that MT predominantly augments Cr tolerance in maize seedlings through the synergistic activation of the antioxidant defense system and the elevation of polyamine levels. Prior research has indicated that polyamines can significantly mitigate the inhibitory effects of Cr toxicity on maize growth by activating the antioxidant defense mechanism and diminishing cellular oxidative damage [20]. Building on these findings, we hypothesize that MT may facilitate the activation of the antioxidant defense system by modulating polyamine metabolism, thereby alleviating Cr toxicity. This hypothesis warrants further exploration through molecular biology methodologies.

## 4. Materials and Methods

### 4.1. Plant Growth and Experimental Treatments

Seeds of the Cr-sensitive maize cultivar “QS101”were first surface sterilized using a 1% hypochlorous acid solution for 10 min and then thoroughly rinsed with sterile water. The treated seeds were evenly distributed on filter paper moistened with distilled water and incubated in darkness at 25 °C for three days to allow germination. Following germination, the seedlings were transplanted into plastic containers containing a 1/2 Hoagland nutrient solution (pH 5.8) for hydroponic cultivation. The maize plants were then maintained in a controlled climate chamber, where day and night temperatures were set at 28 °C and 22 °C, respectively, under a photoperiod of 14 h of light and 10 h of darkness, with a photosynthetically active radiation density of 800 μmol m^−2^ s^−1^. Following a three-week growth period, and based on our prior experimental findings, the leaves were treated with 50 µM melatonin (MT). Subsequently, after 12 h of MT exposure, a concentration of 100 µM potassium dichromate (K_2_Cr_2_O_7_) was added to the nutrient solution to establish Cr stress conditions. The experiment comprised four treatments: Control (no MT, no K_2_Cr_2_O_7_), MT (50 µM MT), Cr (100 µM K_2_Cr_2_O_7_), and MT + Cr (50 µM MT and 100 µM K_2_Cr_2_O_7_). Each treatment condition was replicated across 10 plastic boxes, with each box containing 10 seedlings. Seven days post-Cr stress induction, the roots and leaves of the maize plants were harvested in the morning and stored at −80 °C in an ultra-low temperature freezer for subsequent analyses. Each treatment was conducted with three independent biological replicates.

### 4.2. Measurement of MT and Cr Concentrations and Plant Phenotypic Parameters

The endogenous MT content in maize plants was quantified utilizing high-performance liquid chromatography, as described in reference [54]. An initial 0.5 g sample was accurately weighed and homogenized into a fine powder under liquid nitrogen conditions. Subsequently, 5 mL of methanol was introduced for extraction. The mixture was then centrifuged at 10,000× *g* and 4 °C for 30 min, after which the collected supernatant was dried under a stream of nitrogen gas. The dried residue was reconstituted in 200 μL of a 0.1 M Na_2_HPO_4_–acetonitrile mixture (65:35, *v*/*v*), filtered through a 0.22 μm membrane filter, and a 5 μL aliquot was injected into a C18 chromatographic column (Shimadzu, Kyoto, Japan) for separation. Detection was carried out at a wavelength of 280 nm. The MT content in the sample was determined by referencing a standard curve generated using an MT standard obtained from Sigma-Aldrich (St. Louis, MO, USA). The Cr content in leaves and roots was measured using the method by Hasan et al. [35]. A sample weighing 0.2 g was subjected to digestion using a mixture of HNO_3_ and HClO_4_ in a 5:1 volume ratio at a temperature of 180 °C. The resulting digestion solution was appropriately diluted, and the Cr concentration was measured using an inductively coupled plasma atomic emission spectrometer (ICP-AES, Optima 7000 DV, PerkinElmer, Waltham, MA, USA).

After a 7-day treatment, maize roots and shoots parts were harvested, dried at 80 °C to a constant weight, and their dry weight recorded. The leaf area index was quantified utilizing a LI-3000 leaf area meter (LI-COR, Lincoln, NE, USA). Root vitality was evaluated employing the triphenyl-tetrazolium chloride (TTC) method [55]. For this, root samples weighing 0.1 g were incubated in a solution containing 5 mL of 0.4% TTC and 5 mL of 0.1 M phosphate buffer (pH 7.0) at 37 °C for a duration of 2 h in the absence of light. The extract was then obtained using ethyl acetate, and its absorbance was measured at 485 nm using a UV spectrophotometer (UV-2600, Shimadzu, Kyoto Japan).

### 4.3. Determination of Chlorophyll Content, Fv/Fm and Gas Exchange Parameters

Chlorophyll was extracted from leaves using 95% ethanol, according to Guo et al. [56]. Quantitative analysis was performed using a spectrophotometer. The LI-6800 (LI-COR Biosciences, Lincoln, NE, USA) was used to measure the Pn, Ci, Gs, and Tr of the latest fully expanded leaves between 10:00 and 11:00 a.m. The leaf chamber area was maintained at 2 cm × 3 cm, with a photosynthetic photon flux density set at 1000 μmol m^−2^ s^−1^, relative humidity controlled at 50%, and CO_2_ concentration aligned with ambient conditions. Leaves from the same location as those used for photosynthesis measurements were selected, and following a 20-min period of dark adaptation, the maximum photochemical quantum yield of photosystem II (Fv/Fm) was determined using the PAM2500 chlorophyll fluorescence meter (Walz, Effeltrich, Germany).

### 4.4. Polyamine Content Determination

Based on the procedure described by Flores and Galston [57], different polyamine fractions—including free, soluble-conjugated, and insoluble-bound forms—in maize were analyzed. Briefly, 2 g of frozen samples were ground into a fine powder using liquid nitrogen, followed by extraction with 5 mL of 5% perchloric acid. The homogenate was incubated on ice for 1 h and then centrifuged at 12,000× *g* for 20 min at 4 °C to separate the supernatant from the pellet. The supernatant was used for the determination of free and soluble-conjugated polyamines, whereas the pellet was employed for insoluble-bound polyamine analysis. For free polyamine measurement, 2 mL of the supernatant was combined with 1 mL of 2 M NaOH and 10 μL of benzoyl chloride, followed by incubation at 37 °C for 30 min. Subsequently, 2 mL of saturated NaCl and an equal volume of ether were added, and the mixture was thoroughly vortexed. The mixture was then centrifuged at 4000× *g* for 5 min, and 1 mL of the ether layer was extracted and subsequently dissolved in methanol. After evaporation of the solvent using nitrogen gas and reconstitution in methanol, the sample was subjected to quantitative analysis of Put, Spd, and Spm utilizing a high-performance liquid chromatography (Shimadzu, Kyoto, Japan) system equipped with a C18 reversed-phase column (nanoViper, Thermo Fisher, Waltham, MA, USA).

### 4.5. Analysis of Enzymes Involved in Polyamine Metabolism

The activities of ADC, SAMDC, and ODC were evaluated in accordance with the methodology outlined by Sheteiwy et al. [58]. Frozen samples (0.5 g) were ground and homogenized with 3 mL of extraction buffer (50 mM EDTA, 25 mM potassium phosphate, 100 μM phenylmethylsulphonyl fluoride, 25 mM ascorbic acid (AsA), and 1 mM 2-mercaptoethanol). The homogenate was centrifuged at 25,000× *g* for 20 min at 4 °C, and the supernatant was dialyzed overnight in the extraction buffer. Enzyme activities were evaluated by measuring CO_2_ release. Polyamine-degrading enzyme activities, including DAO and PAO, were determined using a UV-2600 ultraviolet spectrophotometer, as per Wang et al. [16].

### 4.6. Detection of Oxidative Damage and ROS

The quantification of O_2_^−^ was conducted following the protocol outlined in prior research [59]. A 0.5 g sample was mixed with 65 mM phosphate buffer (pH 7.8) and subsequently centrifuged at 5000× *g* for 10 min. The supernatant was then mixed with 50 mM potassium phosphate buffer, 10 mM hydrochloric hydroxylamine, 7 mM naphthylamine, and 17 mM sulfanilamide, and left at 25 °C for 30 min. Absorbance at 530 nm was measured with a spectrophotometer, and O_2_^−^ content was determined using a NaNO_2_ standard curve. The determination of H_2_O_2_ content was performed at a wavelength of 415 nm by measuring absorbance in accordance with the method described by Azarin et al. [60]. The sample was treated with a solution comprising 10% trichloroacetic acid and 0.65% thiobarbituric acid and incubated at 95 °C for 25 min. The concentration of MDA was assessed using the method previously documented [61]. Electrolyte leakage was assessed in accordance with the methodology outlined by Li et al. [62]. In summary, a 0.1 g sample was immersed in 40 mL of ultrapure Milli-Q water and maintained at ambient temperature for a duration of 2 h. The initial electrical conductivity (EC1) was measured using a Mettler Toledo MC-126 conductivity meter. The samples were then boiled for 15 min, allowed to cool to room temperature, and the final conductivity (EC2) was subsequently recorded. Electrolyte leakage was expressed as a percentage and calculated as (EC1/EC2) × 100.

### 4.7. Antioxidant Enzyme Activity Determination

A total of 0.5 g of frozen plant tissue samples was weighed and subsequently homogenized in a pre-cooled extraction buffer. This extraction buffer comprised 0.2 mM EDTA, 2 mM AsA, 25 mM HEPES (pH 7.8), and 2% (*w*/*v*) polyvinylpyrrolidone. It is noteworthy that the extraction buffer utilized for the measurement of SOD activity did not include AsA. The homogenate was subjected to centrifugation at 12,000× *g* for 30 min at 4 °C, after which the supernatant was collected for enzyme activity analysis. SOD activity was assessed following the methodology outlined by Goodarzi et al. [63], through the evaluation of the nitroblue tetrazolium (NBT) reduction reaction, with a detection wavelength set at 560 nm. CAT activity was measured in accordance with the protocol established by Han et al. [64], which involved monitoring the decomposition of H_2_O_2_ at a wavelength of 240 nm. POD activity was determined using the pyrogallol oxidation method [65]. The concentrations of AsA and GSH were quantified in accordance with the methodologies outlined by Lei et al. [66].

### 4.8. Real-Time qPCR Assay

Frozen samples were ground using liquid nitrogen, and total RNA was extracted with a Sangon kit (Beijing, China) following the manufacturer’s protocol. RNA integrity was assessed by gel electrophoresis, and RNA concentration was quantified at 260 nm using a spectrophotometer. Quantitative reverse transcription polymerase chain reaction (RT-qPCR) was conducted utilizing a QuantStudio 1 Real-Time PCR System (Thermo Fisher Scientific, Waltham, MA, USA) with SYBR^®^ Premix Ex Taq^TM^, following the protocol provided by the manufacturer. Primers for the genes of interest were designed utilizing the Primer-BLAST tool (NCBI, Bethesda, MD, USA) available through the National Center for Biotechnology Information (NCBI), with ZmUbi-2 serving as the internal control. Each experimental condition was independently repeated three times. The target gene’s relative expression was assessed via the 2^−△△Ct^ comparative threshold cycle (Ct) approach.

### 4.9. Data Analysis

SPSS software, version 22.0 (IBM, Armonk, NY, USA), was used to conduct the data analysis, while Origin 2024 software (OriginLab, Northampton, MA, USA) was employed for creating graphical representations. Differences among treatments were considered significant at *p* ˂ 0.05.

## 5. Conclusions

**Figure 10:**
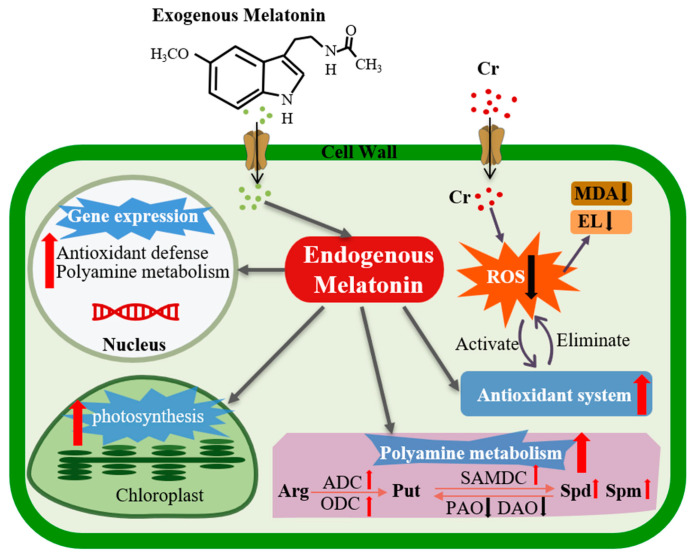
Melatonin alleviates chromium toxicity by promoting polyamine synthesis and activating the antioxidant defense system in maize seedlings.

The findings of this study demonstrate that Cr stress results in the excessive accumulation of ROS and significantly diminishes the levels of photosynthetic pigments and photosynthesis in leaves, thereby severely inhibiting the growth and development of maize. MT, as a multifunctional molecule, mitigates the detrimental effects of Cr on maize growth through various pathways. Specifically, MT enhances antioxidant enzyme activities and increases the levels of non-enzymatic antioxidants, thereby effectively scavenging excess ROS, maintaining redox homeostasis and alleviating Cr-induced inhibition of photosynthesis. Furthermore, MT upregulates the expression of genes involved in polyamine biosynthesis and significantly increases polyamine accumulation, which is closely associated with enhanced maize tolerance to Cr toxicity (Figure 10). Overall, this study provides critical insights into the mechanisms by which MT alleviates Cr toxicity, establishing a theoretical foundation for its sustainable application in agricultural production. Future research will employ transcriptomic and metabolomic approaches to further elucidate the molecular mechanisms through which MT regulates polyamine metabolism to mitigate Cr toxicity.

## Data Availability

The data are contained within the manuscript.
